# Dual-Column HS-GC-FID/FID Method for In-Depth Analysis of Low-Molecular-Weight Volatile Alcohols in *Postmortem* Biological Material

**DOI:** 10.3390/jox16030080

**Published:** 2026-05-06

**Authors:** Paweł Szpot, Olga Wachełko, Kaja Tusiewicz, Marcin Zawadzki

**Affiliations:** 1 Department of Forensic Medicine, Wroclaw Medical University, 4 J. Mikulicza-Radeckiego Street, 50-345 Wroclaw, Poland; 2 Institute of Toxicology Research, Marii Curie-Sklodowskiej 55/61, 50-369 Wroclaw, Poland; 3 Faculty of Medicine, Wrocław Univeristy of Science and Technology, 27 Wybrzeże Wyspiańskiego Street, 50-370 Wroclaw, Poland

**Keywords:** HS-GC-FID/FID, dual columns, Zebron ZB-BAC1, Zebron ZB-BAC2, *postmortem* analysis, tert-butanol

## Abstract

**Aim:** The aim of this study was to develop and validate a reliable HS-GC-FID/FID method for the determination of ethanol and other low-molecular-weight volatile compounds in biological fluids for forensic applications. **Method:** The method is based on headspace gas chromatography with dual-column and dual flame ionization detection (HS-GC-FID/FID), using Zebron ZB-BAC1 and ZB-BAC2 columns. The procedure was validated in terms of linearity, limits of detection and quantification, precision, and accuracy, as well as carryover. **Results:** The method demonstrated linearity over the concentration range of 0.05–5.0‰, with R^2^ values of 0.997–0.999. The limit of quantification (LOQ) was 0.05‰, and the limit of detection (LOD) was 0.025‰. Precision and accuracy were both below 5%. Retention times (min) on the two columns were as follows: methanol (1.70/1.81), ethanol (2.06/2.29), acetone (2.60/2.59), isopropanol (2.45/2.73), n-propanol (3.24/3.98), and n-butanol (6.35/8.45). The developed method was successfully evaluated through international proficiency testing and is routinely applied in our laboratory for forensic casework. **Conclusions:** The developed HS-GC-FID/FID method provides accurate and reliable determination of ethanol and other relevant volatile compounds in biological fluids (i.a., blood) and meets the requirements for forensic toxicology. Additionally, the literature review conducted in this study highlights that globally unified principles for forensic alcohol analysis are still lacking, and that certain inappropriate methodological approaches remain in use. The present paper also provides recommendations defining essential methodological requirements to ensure the evidential reliability of ethanol analysis in forensic toxicology.

## 1. Introduction

The determination of low-molecular-weight volatile alcohols in *postmortem* material using headspace gas chromatography (HS-GC) is routinely performed in forensic laboratories. These analyses are important not only for law enforcement agencies in the broad sense, but also for insurance companies that decide on the payment of compensation under insurance policies. Typically, routine determination of alcohols such as methanol, ethanol, isopropanol, n-propanol, and n-butanol is sufficient to establish whether death occurred as a result of poisoning with these substances. In forensic toxicology practice, however, there are also situations in which the determination of additional chemical compounds in biological material proves to be essential. One such compound is acetone, the determination of which is crucial not only in cases of acetone or isopropanol poisoning (since acetone is a metabolite of the aforementioned alcohol) [1], but also in the diagnosis of ketosis caused by diabetes, hypothermia, or starvation [2]. Criminal cases can be complex, and therefore, to achieve a better understanding of the circumstances preceding death, toxicological analyses are sometimes extended to also include other compounds, such as acetaldehyde [3].

*Postmortem* blood toxicological analysis is of particular importance because it plays a key role in determining the cause of death, especially in cases of unexplained sudden death and deaths occurring under suspicious circumstances. Even a simple screening for volatile chemical compounds can, at an early stage, allow determination of whether death resulted from poisoning or was a consequence of an underlying disease suffered by the deceased (e.g., untreated diabetes) [4].

One of the fundamental assumptions in forensic toxicological investigations is that their results must have evidential value and withstand scrutiny in court. Only such results can be used, for example, to reconstruct the circumstances preceding death, determine the time of substance intake or the dose of a toxic agent, or assess whether the deceased was intoxicated. This is particularly important in cases of traffic accidents, occupational accidents, drownings, domestic violence, and deaths occurring during police custody. It is worth emphasizing that the interpretation of *postmortem* toxicological results requires particular caution due to the multitude of “confounding” factors, such as interindividual variability, substance stability, endogenous formation, the postmortem interval (PMI), and the circumstances of death. To avoid introducing additional variables that could further complicate interpretation, the applied analytical method must be reliable, i.e., accurate, selective, resistant to interference, and preferably verified through independent proficiency testing [5].

In gas chromatographic analysis of low-molecular-weight alcohols, either direct injection or the headspace technique is employed [6]. In the past, even highly invasive approaches were used, such as the direct injection of whole blood into the gas chromatograph injector [7]. However, the headspace technique has become the most widely used technique for the determination of alcohols in forensic laboratories. This preference can be attributed to several well-established advantages, most notably that the method allows the sample to be analyzed with minimal or no preparatory steps, irrespective of the biological matrix (e.g., without extraction or protein precipitation). Additional benefits include practically negligible matrix effects, elimination of the need for inlet liners, thereby minimizing the risk of contamination or carryover within the analytical system, and reduced deterioration of the chromatographic column compared with direct injection.

However, it should be noted that in the context of postmortem samples (particularly those collected from putrefied cadavers), the impact of decomposition on analyte partitioning cannot be precisely assessed. While headspace analysis generally exhibits negligible matrix effects, pre-death specimens from the same individual are rarely available for direct comparison, making it difficult to determine the extent to which postmortem changes might influence measurements. Moreover, the blood samples are subjected to multiple dilutions with an internal standard, which further minimizes potential matrix effects.

Despite numerous established methods for the determination of volatile alcohols in postmortem biological samples, current standard approaches often face limitations that can compromise forensic reliability. Single-column analyses may be prone to co-elution and interferences from structurally similar compounds, while reliance on a single detector increases the risk of undetected fluctuations and errors. Additionally, inappropriate selection of internal standards and postmortem formation of endogenous alcohols and other volatile compounds can further bias quantification. These issues highlight a critical gap, as widely adopted methods that integrate redundant analytical controls with validated procedures to minimize errors and ensure evidential reliability remain limited.

The aim of this study was to develop a method for the determination of methanol, acetone, ethanol, n-propanol, isopropanol, and n-butanol using headspace gas chromatography with dual columns and dual-flame ionization detectors. The practical applicability of the method was demonstrated using authentic forensic samples, including putrefaction fluid and antemortem blood. This study addresses the aforementioned gap by employing a dual-column/dual-FID approach, which allows cross-verification across two independent chromatographic conditions, thereby enhancing both the accuracy and reproducibility of forensic alcohol analysis. Furthermore, this paper aims to draw the attention of toxicologists worldwide to several critical factors that can significantly influence measured ethanol concentrations, potentially leading to legal and financial consequences, such as denial of compensation due to falsely elevated ethanol levels.

## 2. Materials and Methods

### 2.1. Chemicals and Toxicologically Relevant Quality Controls

Water (ChemSolve^®^ LC–MS) was purchased from Witko (Łódź, Poland); tert-Butanol (99.5% purity; internal standard, IS) was purchased from CPAchem (Stara Zagora, Bulgaria); n-propanol (99.8% purity) and n-butanol (99.8% purity) were purchased from CPAchem (Stara Zagora, Bulgaria). Ethanol (99.9%) was purchased from Stanlab^®^ (Lublin, Poland). Methanol (99.9%), isopropanol (99.9%), and acetone (99.5%) were purchased from Sigma-Aldrich (Steinheim, Germany). Due to the limitations of commercially available reference materials, which are typically limited to concentrations of up to 4.0‰, and based on our experience indicating that ethanol intoxications can exceed these levels, we developed our own calibration curve by preparing calibration standards from scratch. This approach was particularly important in cases of severe or fatal intoxications, especially in chronic alcohol users, where blood ethanol concentrations can reach extremely high levels. The aqueous standard solutions of ethanol, methanol, acetone, and isopropanol (used as quality-control samples between batches) were purchased from Cerilliant (Round Rock, TX, USA) in concentrations of 0.25, 0.5, and 2.0‰. For n-propanol and n-butanol (as they are not included in commercial tests), quality controls were created with the use of blank matrix in final concentrations of 0.2 and 0.5‰. Quality-control samples for ethanol in human whole blood, at the legal blood alcohol concentration (BAC) limits for driving under the influence in our country (0.2‰ and 0.5‰, respectively), were obtained from ACQScience (purchased from LGC Standards). Blank blood (without any analytes) was obtained from Medichem (ALC VB 000 Medidrug^®^; Medichem Diagnostica GmbH & Co. Steinenbronn, Germany, purchased from LGC Standards).

### 2.2. Standard Solutions, Working Solutions, and Calibration Standards

The stock solution and standard solutions were stored at 4 °C. Standard solution internal standard (IS), i.e., tert-butanol, was prepared by mixing a pure solution with LC-MS-grade water in a volumetric flask to achieve a concentration of 0.5 mg/mL. Similarly, working solutions of each analyte were prepared by mixing a pure solution of a compound with LC-MS-grade water in a volumetric flask and then diluting such prepared solution with LC-MS-grade water to obtain working standard solutions. Calibration point samples were then prepared by mixing the appropriate working solution with blank whole blood to create concentration levels of 0.05, 0.1, 0.2, 0.5, 1.0, 2.0, 4.0, and 5.0‰.

### 2.3. Sample Preparation Procedure

A volume of 100 µL of biological fluid was added to a headspace vial (10 mL, Alwsci Technologies Shaoxing, Shaoxing, China). Next, 0.5 mL of the IS aqueous tert-butanol solution (0.5 mg/mL) was added. The vials were immediately sealed with headspace caps (aluminum cap: butyl rubber, Polygen, Gliwice, Poland) and vortex-mixed for 5 s.

### 2.4. Apparatus

A Shimadzu GC-2010 Plus AF IVD system (Kyoto, Japan), fitted with an advanced flow controller (AFC), a split/splitless (SPL) inlet, and two flame ionization detectors (FID), was employed. Samples were processed and introduced via a Shimadzu HS-20 static headspace autosampler (Kyoto, Japan), with transfer to the GC through a single SPL inlet. The HS effluent was split equally (1:1) using a SilFlow^®^ microfluidic platform (SHI-980-10593, Trajan, Ringwood, VIC, Australia) and directed onto two capillary columns: Zebron-BAC1 (30 m × 0.32 mm i.d., 1.8 μm film; Phenomenex, Torrance, CA, USA) and Zebron-BAC2 (30 m × 0.32 mm i.d., 1.2 μm film; Phenomenex, Torrance, CA, USA). Each column fed an independent FID channel, allowing concurrent acquisition from both separations. Headspace settings and chromatographic conditions for alcohol congener analysis followed those reported previously in [8]. Operating headspace gas chromatography with dual-column and dual-flame ionization detector parameters are presented in Table 1.

### 2.5. Validation

Validation of the method included examination of selectivity, linearity, precision, and accuracy, carryover limit of quantification (LOQ), and limit of detection (LOD). Validation was performed as described in [9]. Linearity was evaluated by analysis of working solution with human blood in final concentrations of: 0.05, 0.1, 0.2, 0.5, 1.0, 2.0, 4.0, and 5.0‰. Precision (RSD%) and accuracy (RE%) were estimated by analysis of QC samples (*n* = 5) in concentrations of: 0.1, 1.0, and 4.0‰. Intra-day precision and accuracy were assessed using five replicates at each validation level. For intra-day evaluation, all replicates were prepared by a single analyst within one day. Inter-day precision and accuracy were determined over five consecutive days by a different toxicologist, with three samples (low, medium, and high concentrations) analyzed each day. To evaluate potential carryover, three blank samples were analyzed consecutively after the highest-level calibration standard. Carryover was deemed unacceptable if the analyte signal in the blank exceeded the established limit of detection. The limit of quantification (LOQ) was defined as the lowest analyte concentration in the sample matrix that can be determined with acceptable bias (±20%) and precision (RSD ≤ 20%), while the limit of detection (LOD) corresponds to the lowest analyte concentration at which the signal-to-noise ratio is at least 3:1. Each batch was prepared using mandatory quality controls of 0.2‰ and 0.5‰ for ethanol, corresponding to the legal blood alcohol concentration (BAC) limits for driving under the influence in our country, along with one QC for other volatile congeners. In accordance with our routine analytical procedure, each sample was analyzed in duplicate and simultaneously on two chromatographic columns of differing polarity, with the final result calculated as the mean of four measurements. For these analyses, the acceptance criterion of a maximum 5% deviation between results was strictly applied. In cases where the acceptance criterion of a maximum 5% deviation between results is exceeded, additional analyses are routinely performed in our laboratory using GC–MS, and we recommend this approach to verify that no co-eluting substances interfere with the analyte peaks.

## 3. Results

**Figure 1:**
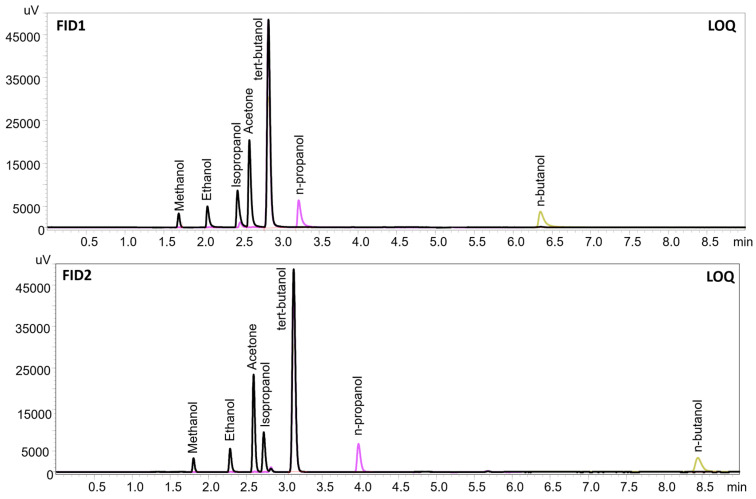
Chromatograms of ISTD and LOQ of volatile compounds obtained from two columns (Zebron ZB-BAC1 and Zebron ZB-BAC2) and two flame ionization detectors. The retention time of tert-butanol (ISTD) on the first column was 2.848, and on the second it was 3.131.

**Figure 2:**
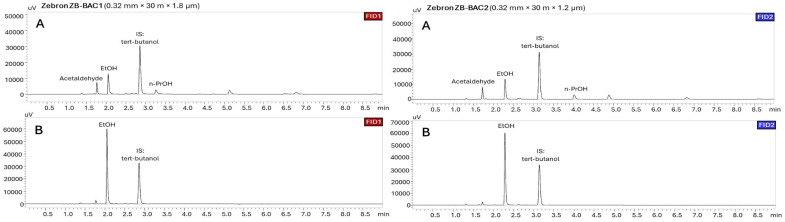
(**A**) Chromatograms of putrefaction fluid (with ethanol concentration of 0.4‰); (**B**) chromatograms of antemortem blood sample (with ethanol concentration of 1.38‰). Results were provided for both columns: Zebron ZB-BAC1 and Zebron ZB-BAC2.

Validation results are shown in Table 2. All presented values fall within the acceptable range for toxicological analysis of biological materials, in accordance with the recommendations of the German Society of Toxicological and Forensic Chemistry (GTFCh) [10]. Quality-control samples were prepared at three concentrations (low, medium, and high relative to the calibration range) by spiking blank samples. Relative error (RE) values within ±15% and precision expressed as relative standard deviation (RSD) ≤ 15% were considered acceptable, in accordance with GTFCh recommendations. The method, developed in 2019, has been subjected to international proficiency testing for ETB Ethanol in blood (ARVECON) and has consistently achieved positive results in each test. Additionally, the developed method for the determination of ethyl alcohol in blood was evaluated and achieved positive results in other proficiency tests, namely: the AXIO LGC Proficiency Testing Scheme in Drugs in blood Quantitative analyte selection, PT-TX-BLD (accreditation ISO/IEC 17043) (LGC Group Limited, Guildford, UK). Furthermore, the HS-GC-FID/FID method has been successfully implemented for routine toxicological analysis in our laboratory during the past 6 years. Chromatograms of all compounds analyzed using the presented method, collected on two columns (Zebron ZB-BAC1 and Zebron ZB-BAC2), are shown in Figure 1. During routine toxicological examinations, we analyzed various biological fluids, including standard matrices such as whole blood, serum, plasma, vitreous humor, and urine, as well as less commonly studied matrices such as bile, putrefaction fluid, and gastric contents. Chromatograms from representative authentic cases are shown in Figure 2, including putrefaction fluid (containing 0.4‰ of ethanol) compared with a standard whole blood sample containing 1.38‰ ethanol.

## 4. Discussion

Ethanol is one of the most widely used psychoactive substances worldwide, primarily due to its legal status in most countries. Heavy alcohol consumption and alcohol dependence represent major public health challenges. According to the World Health Organization, more than 3 million deaths worldwide were attributable to alcohol use or abuse in 2014 [4]. Toxicological analysis of ethanol has become a fundamental laboratory test in forensic toxicology, and ethanol, together with its pharmacokinetics, metabolism, and behavioral effects, seems to be one of the most extensively studied substances in toxicology and the history of intoxications.

In an era dominated by growing concern over novel psychoactive substances, ethanol and other volatile compounds may appear to receive comparatively less scientific attention, giving the impression that their forensic aspects have been exhaustively addressed. This assumption, however, is not supported by current evidence. A substantial number of contemporary forensic studies addressing alcohol analysis continue to be published, as summarized in Table 3. This overview demonstrates that globally unified principles for forensic toxicological analysis of alcohol are still lacking.

Importantly, many of the reviewed analytical approaches are of limited suitability for forensic purposes and should not be considered evidential, despite being presented by the authors as applicable for forensic use. This limitation arises primarily from the inappropriate use of certain volatile compounds, such as n-propanol [12,13,14,17,18] or 3-methyl-2-pentanone [15], as internal standards, despite their known *postmortem* formation [4,20]. As a result of *postmortem* microbial activity, these compounds may be produced endogenously, leading to an unrecognized increase in the internal standard signal. Consequently, ethanol concentrations may be underestimated without the analyst’s awareness. These methodological practices appear to have been adopted from clinical toxicology laboratories, where n-propanol is commonly used as an internal standard. In living individuals, however, its presence is generally unexpected—unless contaminated alcohol has been intentionally consumed. On the other hand, research has shown that n-propanol may also be present in individuals who have consumed ethanol [21,22]. In such cases, these other volatile alcohols may influence analytical outcomes. Despite extensive evidence demonstrating that n-propanol and n-butanol can be formed *postmortem* (similarly to ethanol) through microbial processes, these substances continue to be employed in quantitative ethanol determinations in some forensic studies [4,6].

Moreover, when chromatographic analysis reveals peaks corresponding to acetaldehyde, n-propanol, and/or n-butanol at retention times consistent with these compounds, the measured ethanol concentration in blood should be interpreted with caution, as part or even all of the detected ethanol may result from *postmortem* microbial formation [4]. A recent study from China proposed elevated blood acetaldehyde as a useful biomarker of *postmortem* ethanol synthesis via glycolysis and anaerobic fermentation of pyruvate [3]. This phenomenon is likely illustrated in the chromatogram of putrefaction fluid shown in Figure 2, where these volatile compounds are present alongside low ethanol concentrations. Nevertheless, a comprehensive forensic assessment of alcohol influence must always be conducted in the context of the entirety of the collected evidentiary material in a given case. To illustrate analytical differences using HS-GC-FID/FID, Figure 2 also presents a representative chromatogram of a blood sample collected from a detained driver with a confirmed blood alcohol concentration of 1.38‰.

Regarding the choice of internal standard, a comprehensive study performed by the Federal Aviation Administration in 1998 [23] demonstrated that tert-butanol is the most appropriate compound, as it is not formed *postmortem* (even under extreme conditions such as those encountered in aviation accidents). Additionally, tert-butanol fulfills fundamental analytical criteria for an internal standard, namely chromatographic proximity to the analyte of interest and structural similarity. In contrast, the use of 1,4-dioxane as an internal standard, as reported by Waters et al. (2018) [16], does not meet these requirements. This inadequacy is evident in the published chromatograms, where ethanol and the internal standard exhibit markedly different retention times (around 3.5 min vs. 9.2 min). Although 1,4-dioxane was marked in aviation accidents as generally not being *postmortem*-generated, its analytical suitability remains questionable. Equally concerning is the use of analytical methods that omit an internal standard. Such approaches lack control over the pre-analytical as well as analytical process and rely solely on external calibration curves, which are inherently less stable and less accurate. Given the legal significance of ethanol determinations, this practice should be considered unacceptable in forensic laboratories.

The use of tert-butanol as an internal standard has been reported previously; however, those studies employed a single chromatographic column [11,12,16,17,18]. Situations have been documented in which unexpected interferences appeared exclusively on one column, such as, for example, sevoflurane co-eluting with ethanol [24]. Such interference may result in falsely elevated ethanol concentrations, potentially leading to serious legal consequences, particularly when results fall near statutory thresholds. When only a single chromatographic column is used, such interferences may remain entirely undetected.

Furthermore, helium was selected as the carrier gas for this study due to its better chromatographic performance compared to nitrogen. Helium provides higher optimal linear velocity and improved efficiency, resulting in sharper peaks, better resolution, and shorter analysis times. In contrast, nitrogen, while more cost-effective, can lead to broader peaks and reduced separation efficiency, particularly for complex mixtures of volatile organic compounds (that can be found in, e.g., postmortem samples). Given the focus on forensic reliability and the need for precise quantification, helium was chosen from the outset to maximize method sensitivity, reproducibility, and robustness.

The application of two chromatographic columns with different polarities significantly enhances analytical selectivity and process control. Notably, only one previously published study (similar to the one in this paper) received two positive evaluations in Table 3. Although the authors employed a single column, the use of mass spectrometric detection provided additional selectivity by introducing mass spectral identification [19]. Deuterated analogs of alcohols represent an optimal solution for quantitative determinations in such systems. Nevertheless, GC–MS, like any analytical approach, has inherent limitations. While it offers superior selectivity and specificity compared to FID and represents a more advanced analytical technique, it is also more complex, costly, and particularly sensitive to matrix effects, ion source contamination, and temporal signal degradation. High-throughput analyses require frequent maintenance and careful monitoring of ionization efficiency to prevent sensitivity loss. In scenarios where analytical simplicity and high throughput are desired, a dual-column system coupled with dual FID detection may therefore represent the most practical and robust solution.

It is also worth noting that the method described herein, owing to its broad analytical capabilities and suitability for routine laboratory implementation, enabled the detection of a non-routine volatile compound, namely chloroform, in an authentic intoxication case [25]. This demonstrates that the broad and comprehensive analysis enables a more comprehensive diagnosis of poisoning and its circumstances.

## 5. Conclusions

The findings presented in this study demonstrate that reliable forensic determination of ethanol and other volatile compounds requires a comprehensive analytical strategy that extends beyond routine instrumental analysis. Despite the long-standing use of alcohol determinations in forensic practice, significant methodological limitations persist, particularly with respect to selectivity, internal standard selection, and control of pre-analytical and analytical variables. Based on a critical evaluation of currently published methods and supported by experimental evidence and authentic forensic casework, several key methodological principles emerge as essential for ensuring the evidential reliability of ethanol analysis in forensic toxicology. These principles are summarized below:

- Using two columns with different polarities combined with two detectors.
- Avoiding internal standards such as n-propanol, n-butanol, and other substances that are formed *postmortem* in forensic investigations.
- Utilizing tert-butanol as an internal standard in forensic toxicological practice.
- Analysis of quality-control (QC) concentrations defined by law for DUI threshold limits, with verification that precision and accuracy remain within acceptable ranges.
- Ensuring that sample preparation is performed in facilities free from exposure to airborne organic solvent vapors, particularly methanol and ACN (which are routinely used for protein precipitation in other toxicological analyses).
- Preparation of analytical batches including two replicates of each sample, analyzed in a forward- and reverse-order sequence (e.g., samples 1–5 followed by 5–1) to verify analytical consistency.
- Verification of internal standards and blank samples in each batch to ensure the absence of contamination during the pre-analytical phase.
- Ensuring compliance with the acceptance criterion of a maximum 5% deviation between results, based on four measurements per sample.
- Careful chromatographic evaluation and critical assessment of peak shape, symmetry, and resolution to minimize the risk of undetected interferences, particularly when using detectors such as FID, where retention time is the sole discriminating parameter.
- Routine performance of independent proficiency tests to assess whether the method meets required accuracy and precision standards.
- Biological fluids intended for forensic toxicology testing should be collected in tubes containing sodium fluoride (NaF) and stored under controlled conditions in a refrigerator maintained at 2–8 °C.

## Figures and Tables

**Table 1:** Summarization of dual column HS–GC–FID/FID operating parameters.

Headspace Parameters		GC Parameters	
Oven temperature	65 °C	Carrier gas	He
Sample line temperature	150 °C	First column	Zebron ZB-BAC1 0.32 mm × 30 m × 1.80 µm
Transfer line temperature	150 °C	Second column	Zebron ZB-BAC2 0.32 mm × 30 m × 1.20 µm
Shaking level	1.0	Column temperature	40 °C
Multi-injection count	1.0	Pressure	80.1 kPa
Pressurize gas pressure	60 kPa	Total flow	55.1 mL/min
Equilibrating time	10 min	Column flow	2.57 mL/min
Pressurizing time	0.5 min	Linear velocity	40.0 cm/sec
Pressure equlib. time	0.1 min	FID1 and FID 2 temperature	240 °C
Load time	0.5 min	FID1 and FID 2 makeup flow	30 mL/min
Load equilibrating time	0.1 min	FID1 and FID 2 H2 flow	40 mL/min
Injection time	1.0 min	FID1 and FID 2 air flow	400 mL/min
Needle flush time	1.0 min	APC1 pressure	60 kPa
Injection mode	Split	Total program time	9.0 min
Sampling time	1.0 min		

**Table 2:** Validation results for six quantified volatile compounds: methanol, ethanol, acetone, isopropanol, *n*-propanol, and *n*-butanol.

	Coefficient of Determination (*R*^2^)		Retention Time [min]		Concentration [‰]	Intra-Day Validation Results		Inter-Day Validation Results		LOQ	LOD
Substance	FID-1	FID-2	FID-1	FID-2		Precision (RSD%)	Accuracy (RE%)	Precision (RSD%)	Accuracy (RE%)		
**Methanol**	0.999	0.999	1.70	1.81	0.1	4.5	1.0	4.0	0.0	0.05‰	0.025‰
					1.0	2.0	1.5	2.0	1.0		
					4.0	1.5	3.5	2.5	5.0		
**Ethanol**	0.999	0.999	2.06	2.29	0.1	2.5	2.0	3.0	2.0	0.05‰	0.025‰
					1.0	1.0	3.0	2.0	0.5		
					4.0	1.0	4.0	3.5	5.0		
**Acetone**	0.999	0.999	2.60	2.59	0.1	1.0	4.5	0.5	3.0	0.05‰	0.025‰
					1.0	0.0	5.0	0.5	5.0		
					4.0	1.5	4.0	3.5	3.0		
**Isopropanol**	0.999	0.999	2.45	2.73	0.1	2.0	3.0	3.0	3.0	0.05‰	0.025‰
					1.0	1.0	4.0	1.0	4.0		
					4.0	1.0	1.0	2.0	4.0		
***n*** **-propanol**	0.997	0.998	3.24	3.98	0.1	1.0	2.5	2.0	3.0	0.05‰	0.025‰
					1.0	1.0	2.0	2.0	4.5		
					4.0	2.0	4.5	1.5	5.0		
***n*** **-butanol**	0.999	0.999	6.35	8.45	0.1	1.5	4.5	3.5	1.5	0.05‰	0.025‰
					1.0	0.0	2.0	4.0	4.0		
					4.0	3.0	4.5	3.5	3.0		

RSD—relative standard deviation; RE—relative error; LOQ—limit of quantification; LOD—limit of detection.

**Table 3:** Summarization of modern methods for determination of volatile compounds in *postmortem* samples utilizing gas chromatography.

No	Method		Sample Volume	Carrier Gas	Column(s)		Internal Standard		Year	Refs.
**1.**	HS-GC-FID	•	1 mL	N_2_	Elite 624 (75 m × 0.53 mm i.d. × 3.0 µm)	•	*tert*-butanol	•	2008	[11]
**2.**	GC-FID (direct injection)	•	100 µL	He	CPWax 57 CB (25 m × 0.25 mm i.d. × 0.2 µm)	•	*n*-propanol	•	2009	[12]
**3.**	HS-GC-FID/FID	•	100 µL	N_2_	RTX-BAC1 (30 m × 0.53 mm i.d. ―) RTX-Bac-2 (30 m × 0.53 mm i.d. ―)	•	*n*-propanol	•	2011	[13]
**4.**	GC-FID/MS	•	100 µL	He	DB-ALC1 (30 m × 0.32 mm i.d. × 1.8 µm) ― (2.89 m × 0.18 mm i.d. ―)	•	*n*-propanol	•	2011	[14]
**5.**	HS-GC-FID/FID	•	100 µL	N_2_	RTX-BAC1 (30 m × 0.32 mm i.d. × 1.8 µm) RTX-BAC2 (30 m × 0.32 mm i.d. × 0.6 µm)	•	3-methyl-2-pentanone	•	2015	[15]
**6.**	HS-GC-FID	•	200 µL	He	RTX-1 (60 m × 0.32 mm i.d. × 3.0 µm)	•	1,4-Dioxane	•	2018	[16]
**7.**	HS-GC-FID	•	1 mL	He	DB-5 MS Elite-1701 (30 m × 0.53 mm i.d. × 1.0 µm)	•	*n*-propanol	•	2020	[17]
**8.**	HS-GC-FID	•	50 µL	He	HP-Innowax (30 m × 0.25 mm i.d. × 0.25 µm)	•	*n*-propanol	•	2020	[18]
**9.**	HS-GC-MS (SIM)	•	100 µL	He	DB-BAC1 Ultra Inert (30 m × 0.32 mm i.d. × 1.8 µm)	•	Deuterated analogs ^a^	•	2025	[19]
**10.**	HS-GC-FID/FID	•	100 µL	He	Zebron-BAC1 (30 m × 0.32 mm × 1.8 µm) Zebron-BAC2 (30 m × 0.32 mm × 1.2 µm)	•	*tert*-butanol	•	2026	**Presented** **method**

**Methods were selected based on the purpose of their use in forensic toxicology routine caseworks declared by the authors**; ― indicates that information was not provided; green color—the appropriate condition for forensic analysis; red color—parameter that should be avoided in forensic testing; gray color—indicates that the choice is acceptable but could be improved; ^a^ Deuterated ethanol, acetone, 2-propanol, and 1-butanol.

## Data Availability

The data supporting the findings of this study (including raw data) are available from the corresponding author upon reasonable request. Access to certain data may be restricted due to confidentiality requirements related to forensic casework and protection of proprietary information.
